# Profiles of telomeric repeats in Insecta reveal diverse forms of telomeric motifs in Hymenopterans

**DOI:** 10.26508/lsa.202101163

**Published:** 2022-04-01

**Authors:** Yihang Zhou, Yi Wang, Xiao Xiong, Arthur G Appel, Chao Zhang, Xu Wang

**Affiliations:** 1 Fundamental Research Center, Shanghai YangZhi Rehabilitation Hospital (Shanghai Sunshine Rehabilitation Center), School of Life Sciences and Technology, Tongji University, Shanghai, China; 2 Department of Pathobiology, College of Veterinary Medicine, Auburn University, Auburn, AL, USA; 3 Auburn University Center for Advanced Science, Innovation, and Commerce, Alabama Agricultural Experiment Station, Auburn, AL, USA; 4 Ministry of Education Key Laboratory of Contemporary Anthropology, Department of Anthropology and Human Genetics, School of Life Sciences, Fudan University, Shanghai, China; 5 Human Phenome Institute, Fudan University, Shanghai, China; 6 Department of Entomology and Plant Pathology, Auburn University, AL, USA; 7 HudsonAlpha Institute for Biotechnology, Huntsville, AL, USA

## Abstract

Telomeres consist of highly conserved simple tandem telomeric repeat motif (TRM): (TTAGG)n in arthropods, (TTAGGG)n in vertebrates, and (TTTAGGG)n in most plants. TRM can be detected from chromosome-level assembly, which typically requires long-read sequencing data. To take advantage of short-read data, we developed an ultra-fast Telomeric Repeats Identification Pipeline and evaluated its performance on 91 species. With proven accuracy, we applied Telomeric Repeats Identification Pipeline in 129 insect species, using 7 Tbp of short-read sequences. We confirmed (TTAGG)n as the TRM in 19 orders, suggesting it is the ancestral form in insects. Systematic profiling in Hymenopterans revealed a diverse range of TRMs, including the canonical 5-bp TTAGG (bees, ants, and basal sawflies), three independent losses of tandem repeat form TRM (Ichneumonoids, hunting wasps, and gall-forming wasps), and most interestingly, a common 8-bp (TTATTGGG)n in Chalcid wasps with two 9-bp variants in the miniature wasp (TTACTTGGG) and fig wasps (TTATTGGGG). Our results identified extraordinary evolutionary fluidity of Hymenopteran TRMs, and rapid evolution of TRM and repeat abundance at all evolutionary scales, providing novel insights into telomere evolution.

## Abstract (precis)

A bioinformatic pipeline for telomeric repeat motif detection was applied to 129 insect species and identified diverse forms of telomeric motifs in Hymenopterans, including canonical 5-bp, novel 8- and 9-bp forms, suggesting extraordinary evolutionary fluidity of telomeric repeat in Hymenopterans.

## Introduction

Telomeres are vital and highly conserved DNA–protein complexes located at the ends of eukaryotic chromosomes that protect chromosome ends from deterioration or fusion with neighboring chromosomes. During DNA replication, an RNA primer is required by the polymerase, and the lagging strand at the chromosomal ends cannot be replicated, shortening the chromosome by 50∼100 bp per division in normal diploid human cells (Wright et al, 1997; Chow et al, 2012). To solve this issue, an RNA-dependent DNA polymerase called telomerase can restore the telomere length by adding telomeric sequences using an RNA template. Telomere dynamics and regulation play critical roles in aging, cancer, cell proliferation, and gene repression (Blackburn, 1991, 2000; Zakian, 1995; Verdun & Karlseder, 2007). Misregulation of telomeres may lead to senescence and genomic instability (Greider, 1996; Sahin et al, 2011).

**Figure 1.**
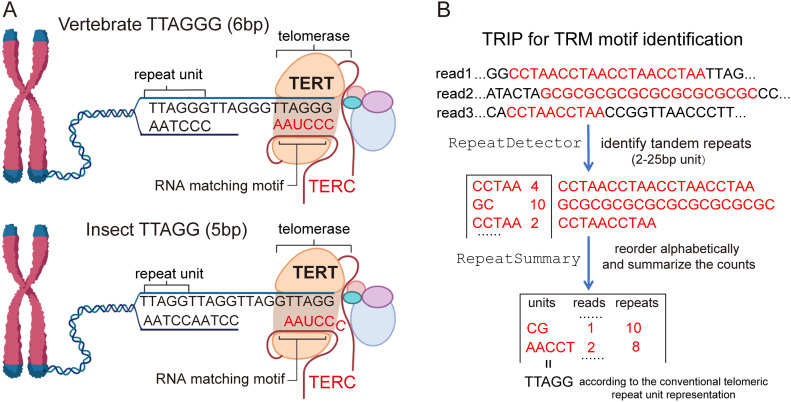
A bioinformatic pipeline to identify short tandem repeats using whole-genome sequencing data. **(A)** A schematic model of telomere maintenance. The telomerase RNA component (TERC) pairs with the telomeric DNA unit (in black) to form an RNA/DNA duplex at the active site of the telomerase reverse transcriptase (TERT). TERT catalyzes the addition of telomeric repeats based on the seed sequence in the TERC template region (5′-TTAGG-3′ in insects and 5′-TTAGGG-3′ in vertebrates). **(B)** Workflow of RepeatMaster for simple tandem repeat detection. RepeatDetector scans through short-read sequencing data in FASTQ format and identifies all short tandem repeats 2–25 bp in length with zero-mismatch tolerance. RepeatSummary reorders the repeat units alphabetically and summarizes the repeat regions into count tables. In this example, the AACCT units are from the telomeric regions containing TTAGG (reverse complement). The convention is to organize the telomeric repeat motif monomer ending with consecutive Gs.

In most eukaryotic organisms, telomeres are comprised of G-rich short tandem telomeric repeat motif (TRM). For example, human telomeres are composed of ∼230 Kb region of 6-bp (TTAGGG)n TRM (de Lange, 2004). TRM is highly conserved in animals (Moyzis et al, 1988; Meyne et al, 1989). All vertebrates examined share the same 6-bp TRM (TTAGGG)n and are believed to evolve from a common ancestor over 400 million years ago (Mya) (Meyne et al, 1989; Traut et al, 2007). This high-level TRM sequence conservation is presumably driven by the conserved TRM binding machinery and the telomerase RNA component (TERC). TERC is an RNA component of the telomerase ranging from 312 to 559 nucleotides in size in vertebrates (Logeswaran et al, 2021) and serves as the template for telomere elongation (Counter et al, 1998; Chen et al, 2000; Lundblad, 2002) (Fig 1A). In mammals, six telomere-associated proteins (TRF1, TRF2, POT1, TIN2, TPP1, and RAP1) form a complex named Shelterin, which is an essential protein complex mediating telomere protection (de Lange, 2018).

TRM variants were found in single-cell eukaryotes and basal invertebrates, such as (TTGGGG)n in *Tetrahymena* and (TTAGGC)n in *Caenorhabditis elegans* (Zakian, 1995). Plant TRMs are more variable and, in some cases, longer than 6-bp (Peska & Garcia, 2020). A 7-bp TRM (TTTAGGG)_n_ was discovered in *Arabidopsis* (Richards & Ausubel, 1988) and *Nicotiana tabacum* (Fajkus et al, 1995), which is the common type found in 44 species belonging to 14 families of angiosperms, gymnosperms, and bryophytes (Fuchs et al, 1995). Many Asparagus species have the vertebrate-type 6-bp (TTAGGG)n, and (TTGGGG)n were also found in this order (Sykorova et al, 2003). Two intermingled TRM variants (TTCAGG and TTTCAGG) were reported in the carnivorous genus *Genlisea* (Tran et al, 2015). An unusually long telomeric sequence (TTTTTTAGGG)n was discovered in *Cestrum elegans* (Peska et al, 2015), and an even longer TRM (TTATGGGCTCGG)n was reported in Allium plants (Fajkus et al, 2016). The simple tandem repeat is not the only form of telomere composition. *Drosophila* is known to have a telomerase-independent mechanism to maintain chromosome integrity (Lundblad, 2002; Pardue & DeBaryshe, 2003; Mason et al, 2008, 2016), which lacks TRM.

In insects, (TTAGG)n (Menezes et al, 2017) was found to be the major TRM in multiple species/orders (Vitkova et al, 2005), including silkworms (Lepidoptera) (Okazaki et al, 1993), ants (Hymenoptera) (Lorite et al, 2002), northern caddisflies (Trichoptera) (Frydrychová et al, 2004), scarabs (Coleoptera) (Sahara et al, 1999), alderflies (Megaloptera) (Frydrychová et al, 2004), and others. A few motif sequence variations, such as (TCAGG)n, were discovered in Coleoptera (Mravinac et al, 2011). The canonical (TTAGG)n was not observed in the parasitoid wasps (Werren et al, 2010; Gokhman et al, 2014), except for the recent report of an 8-bp TRM in the jewel wasp species *Nasonia vitripennis* (Dalla Benetta et al, 2020). Some insects, such as *Drosophila* (Villasante et al, 2007; Saint-Leandre et al, 2019) and Dipteran (Lopez et al, 1999; Gorab, 2003), are known to lack TRM. Therefore, insects exploit diverse mechanisms for telomere solutions during evolution.

The major limitation in studying insect telomeres is the availability of a fast and accurate detection method. FISH and other hybridization approaches were used to confirm TRMs, but they failed in finding novel TRMs. First, hybridization probes are synthesized to target TTAGG, TTAGGG, or TTTAGGG repeat, which will not bind to novel repeat variants with sequence mismatches. Second, the FISH protocol needs to be optimized for different species (Spence et al, 1998; Sahara et al, 1999; Frydrychova & Marec, 2002; Osanai et al, 2006; Mravinac et al, 2011; Novotna et al, 2011; Golub et al, 2015, 2018; Kuznetsova et al, 2017), resulting in false negatives in TRM detection. Last, a negative result is not sufficient to suggest a lack of TRM, which could be due to limited resolution to detect short telomeres. Recently, telomere-to-telomere quality genome assemblies have emerged, thanks to the PacBio and Oxford Nanopore long-read sequencing technologies, which allow direct identification of TRM by examining the chromosome ends. However, the results depend on the assembly quality at the chromosome termini, and it is not currently cost-effective to perform long-read assembly for every insect genome. To leverage the existing short-read sequencing data, a bioinformatic pipeline for TRM identification is urgently needed to explore insect TRM diversity.

A bioinformatic pipeline was successfully applied by Melters et al (2013) for centromere research. Bioinformatics tools have also been demonstrated to facilitate telomere identification (Nersisyan & Arakelyan, 2015; Somanathan & Baysdorfer, 2018; Feuerbach et al, 2019; Harris et al, 2019; Alaguponniah et al, 2020), but these tools are not designed specifically for TRM discovery. Another pipeline called BAL31-NGS had been demonstrated to successfully identify unknown TRM (Peska et al, 2017), but a wet-lab BAL31 digestion experiment was required and cannot be applied to existing short-read data. In this research, we developed an ultra-fast and accurate bioinformatic pipeline named Telomeric Repeats Identification Pipeline (TRIP). The input is short-read whole-genome sequencing (WGS) data with a minimum depth of 10, which is readily available from public databases and can be obtained from genome resequencing. TRIP provides de novo TRM identification, which is not limited by prior knowledge of candidate motifs. Negative results from TRIP are also meaningful, indicating potential loss of simple tandem repeat as TRM and suggesting a complex form of telomere composition, such as collections of retrotransposons in *Drosophila* (Levis et al, 1993). We applied this pipeline to explore the predicted TRMs of 129 species, primarily focusing on TRM evolution in Insecta. Our methodology will facilitate TRM discovery and will shed light on the telomere function and evolution.

## Results

### TRIP, an ultra-fast and precise bioinformatic pipeline for TRM identification

TRMs have a high abundance among the short tandem repeats in the genome. According to this feature, we developed a TRIP for TRM identification from Illumina short-read genome sequencing data by profiling short tandem repeats in sequence reads (Fig 1B). Specifically, RepeatDetector is used to extract 2–25-bp short tandem repeat sequences from Illumina reads. Based on the results, RepeatSummary will reorder the repeat monomers alphabetically, count the total number of repeat-containing reads, and compute the number and the total length for each repeat unit (Fig 1B). TRIP outputs 19 summary statistics and makes inferences of candidate TRMs, based primarily on the number of repeats containing reads, the total tandem repeat length, and the percent of repeat region in the sequencing reads (Table S1). A final TRM is called as the single best candidate with threefold or higher abundance than other repeat units (Table S2). If this condition is not met, the single best candidate will be evaluated by additional evidence from experimental results in the literature or high-quality genome assembly to make the final TRM call (Table S2). We refer the final TRM calls from the TRIP pipeline as predicted TRMs.

Table S1 Telomeric repeat motif candidates identification in Telomeric Repeats Identification Pipeline.

Table S2 Telomeric repeat motif calling criteria in Telomeric Repeats Identification Pipeline.

To evaluate the performance, we applied the TRIP pipeline on 91 species with TRM information reported in the literature across four phyla and nine classes, including plants, vertebrate and invertebrate species (Supplemental Data 1). For the 41 species with simple tandem type TRMs, TRIP correctly identified all of them from short-read data (Supplemental Data 2). The remaining 50 species are known to have retrotransposon/complex telomeres (e.g., Dipterans) or lack TTAGG/TTAGGG TRMs (Supplemental Data 1). The TRIP pipeline determined the absence of any simple tandem repeat type TRM repeat motifs for 50 species (Supplemental Data 3). For the 91 species, TRIP has 100% accuracy and zero false positives. The average computational processing speed is 10 Kbp of sequencing read data per second per process when run on a Linux machine with Intel Xeon Processor E5-2660 CPU at 2.20 GHz, with negligible memory cost.

### Independent identification and confirmation of a previously reported (TTATTGGG)n TRM in a jewel wasp species *N. vitripennis* (*Nv*)

**Figure 2.**
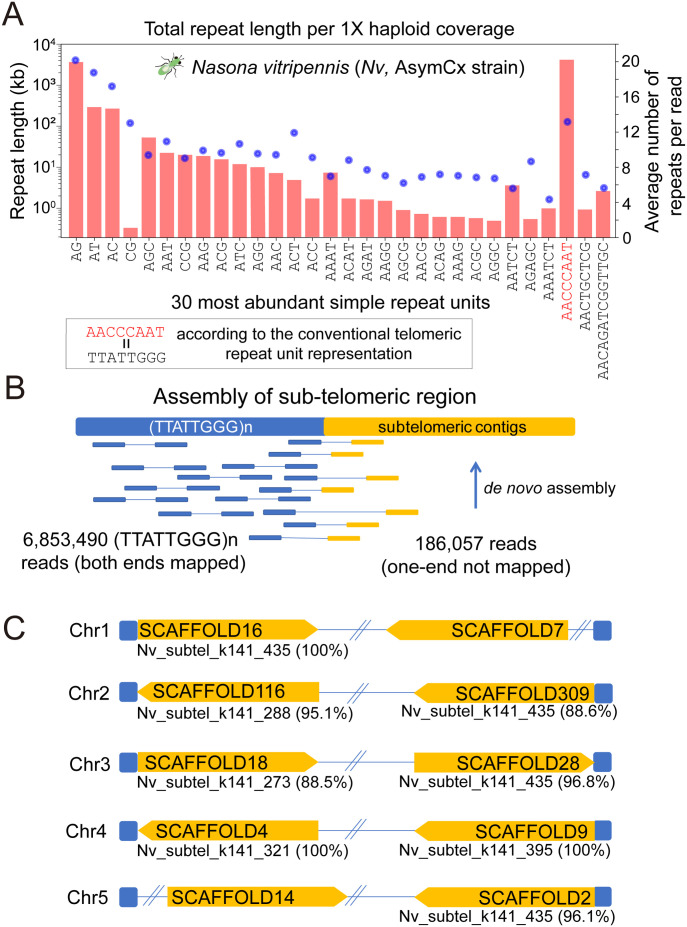
Identification and confirmation of 8-bp telomeric repeats in the jewel wasp *Nasonia vitripennis* (*Nv*). **(A)** Top 30 repeat motifs of total repeat length per 1× haploid genome on the *x*-axis. The red bars represent the total repeat length (log_10_ scale). The blue dots are the average number of repeats per read. The identified telomeric repeat motif (AACCCAAT)n is labeled in red. According to the convention in telomeric research, the telomeric repeat motif is represented in the form of TTATTGGG (reverse complement). **(B)** De novo assembly of the telomeric region in *Nv*. Among 6,853,490 read pairs mapped to the (TTATTGGG)n repeat region, 186,057 only have one read mapped to the telomeric region. The unmapped reads were assembled into sub-telomeric contigs. **(C)** Assembled sub-telomeric regions were aligned to *Nv* reference genome assembly v2.1, and they were mapped to eight chromosomal end scaffolds. Sequence identities between sub-telomeric regions and *Nv* scaffolds were labeled for each hit.

In 2020, Dalla Benetta et al (2020) assembled the *Nv* PSR strain using PacBio and Oxford Nanopore long-read technologies, with significantly improved genome completeness (Dalla Benetta et al, 2020). The authors observed TTATTGGG repeats at chromosome ends and confirmed the chromosomal termini location using FISH in the PSR strain (Dalla Benetta et al, 2020). This was much longer than the known TRMs in insects or other animals. We independently identified (TTATTGGG)n TRM in a different *Nv* strain, AsymCx, using the TRIP pipeline with 125.8 Gbp of 10× Genomics genome resequencing data (Fig 2A). With 426.2× genome coverage, we performed comprehensive profiling of short tandem repeats in the *Nv* genome. In fact, (TTATTGGG)n is the most abundant short tandem repeat in *Nv* (Fig 2A), accounting for 1.4% of the genome with over 4,111 Kbp in length per haploid genome. We examined its genomic locations by assembling the potential sub-telomeric contigs using pair-ended reads with only one repeat mapped to the TRM (Fig 2B). The sub-telomeric contigs map to 8/10 chromosome ends (2*n* = 10) in the *Nv* assembly v2.1 of the AsymCx strain (National Center for Biotechnology Information [NCBI] accession number https://www.ncbi.nlm.nih.gov/assembly/GCA_000002325/), confirming that they are truly the TRM (Fig 2C and Table S3). In the Dalla Benetta et al (2020) PacBio assembly (https://www.ncbi.nlm.nih.gov/assembly/GCA_009193385/), the TTATTGGG type of motif was found at 7 of 10 chromosome ends, totaling 21 Kbp in length (Table S4). This number is much less than the TRIP estimate (over 4 Mbp) because the long simple repeat sequences are difficult to assemble, especially at the chromosome ends.

Table S3 *Nasonia vitripennis* genome mapping results of TTATTGGG neighboring sub-telomeric region assembly.

Table S4 Telomeric length and number telomeric repeat motifs in the *Nv* PSR genome assembly.

### TTAGG is the ancestral TRM across 19 Insecta orders

After confirming the unprecedented long-form TRM (TTATTGGG)n, we collected 7 Tbp whole-genome short-read resequencing data of 129 Insecta species across 21 orders (117 from publicly available databases and 12 generated in this research, see Supplemental Data 4). The remaining 10 insect orders were not included in this analysis due to a lack of data (Table S5). If the chromosome terminal location of the predicted TRM was supported by experimental evidence or chromosome-level genome assembly, the reference was cited and discussed (Supplemental Data 4). If the terminal position was not demonstrated by experimental support for the identified TRMs, the TRIP call was referred to as predicted TRM (Supplemental Data 4).

**Figure 3.**
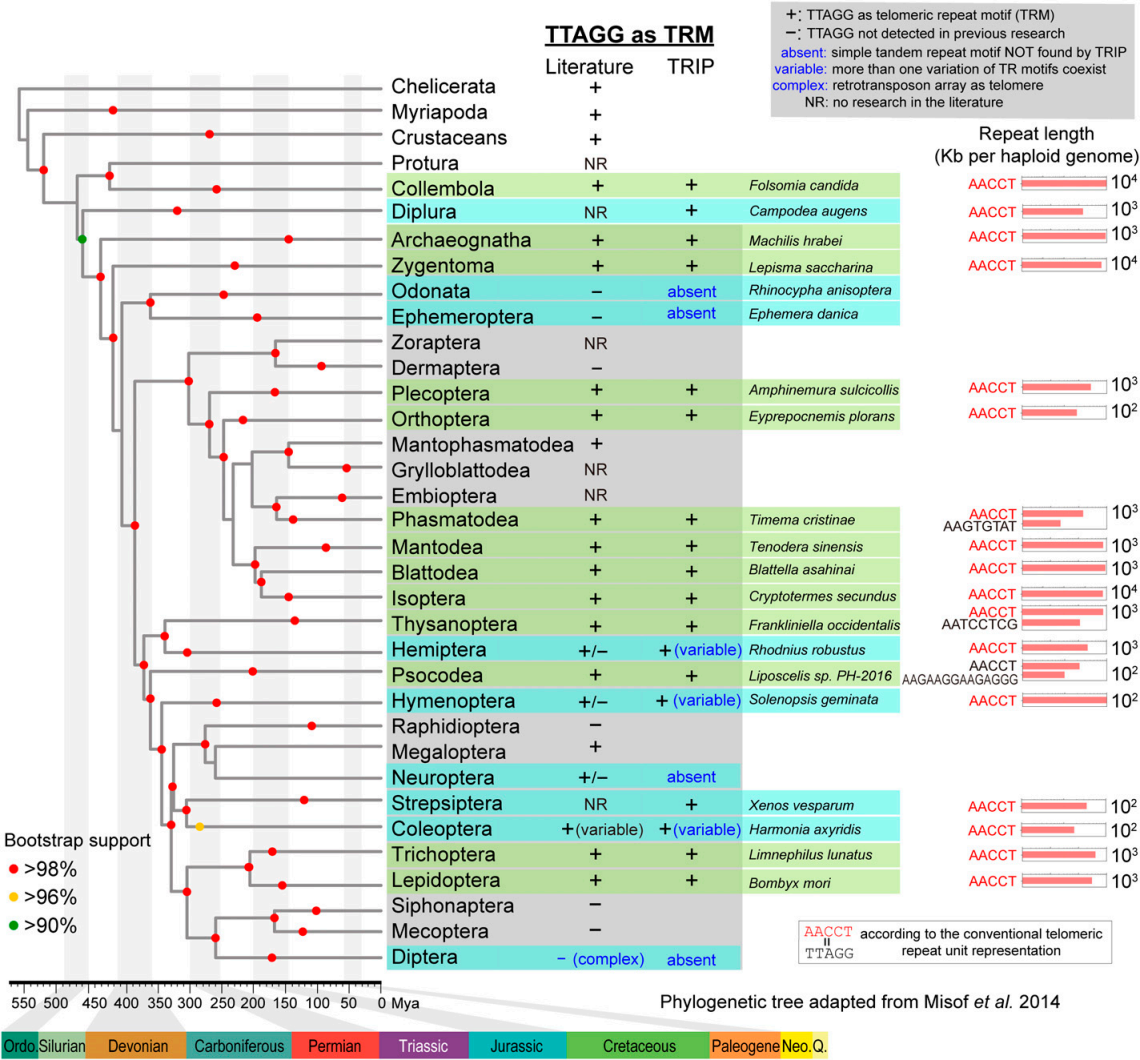
(TTAGG)n is the common and ancestral telomeric repeat motif (TRM) in Insecta. TRM profiles for insects (shaded) and arthropod outgroup species (nonshaded) summarized on the Insecta phylogeny adapted from Misof et al (2014). Previous reports of presence (+) or absence (−) of (TTAGG)n were listed under the literature column. TRM calls from Telomeric Repeats Identification Pipeline (TRIP) in this study are represented by “+” sign or the “absent” label (no tandem repeat TRM form observed). The “variable” label stands for novel TRM variants found. The branches are shaded in green if the TRIP results agree with previous literature reports in all species within this taxon. The branches are shared in blue if novel TRM information is provided by TRIP. Branches without genomic data are shaded in gray. For TTAGG “+” families identified by TRIP, the total repeat lengths are shown in horizontal bar plots for one representative species per branch.

Table S5 Major Insecta order and Hymenopteran superfamily with no or insufficient sequencing data for telomeric repeat motif call.

(TTAGG)n motifs were reported to be the TRM in non-insect arthropod lineages, such as Chelicerata (spiders, tick, etc.) (Vitkova et al, 2005), Myriapoda (millipedes, centipedes, etc.) (Vitkova et al, 2005), and Crustaceans (crabs, lobsters, etc.) (Vitkova et al, 2005). Both Insecta and Entognatha belong to epiclass Hexapoda, and we examined the three sister groups in Entognatha. (TTAGG)n was previously found in Collembola (springtails) by a staining method (Vitkova et al, 2005). We independently confirmed this finding in *Folsomia candida*. No telomere research was reported in Diplura, and we discovered (TTAGG)n as the predicted TRM in *Campodea augens* (Supplemental Data 4–6), suggesting this 5-bp unit is the ancestral form of TRM in arthropods (Fig 3).

For the 129 insect species we investigated, 110 candidate TRMs were discovered in 63 species by TRIP according to the criteria summarized in Table S1. Among these 63 species, 78% (49/63) have a single candidate TRM (Supplemental Data 6), and the TRM was called without additional filtering criteria in Table S2. Previous studies of insect telomere have determined the presence, as well as chromosomal locations of (TTAGG)n and (TTAGGG)n using FISH probes (results summarized in Fig 3). (TTAGG)n was found as the TRM in at least one species of 18 orders. Among these 18 orders with known (TTAGG)n telomere, we identified the (TTAGG)n TRM in 16 of them (Fig 3 and Supplemental Data 4) except for Neuroptera (no simple tandem repeat type TRM found) and Mantophasmatodea (lack of data). No previous telomere research was reported in Strepsiptera, and we identified (TTAGG)n as the predicted TRM for the first time in *Xenos vesparum* (Fig 3 and Supplemental Data 4). We also performed whole-genome sequencing of *Lepisma saccharina*, which is a living fossil in the basal order Zygentoma (see the Materials and Methods section). (TTAGG)n motif was found as the predicted TRM, although a lack of telomerase activity was reported in this silverfish species (Korandova et al, 2014). Collectively, (TTAGG)n was found in 19 insect orders, indicating it is the ancestral form of telomeric tandem repeat in insects.

Dipterans, including fruit flies and mosquitos, have lost the TRM. Instead, they use retrotransposon elements as telomeres to protect the chromosomal ends from deterioration. We examined 16 Dipteran species, and all of them lack a tandem repeat type predicted TRM (Supplemental Data 6). Among them, *Drosophila melanogaster* has 13, *Aedes albopictus* has 20 candidate motifs (Table S6), and none of them were qualified using the TRIP TRM calling requirements (Supplemental Data 6). The TRM in orders Odonata and Ephemeroptera is still unknown, and previous research confirmed the lack of (TTAGG)n (Frydrychova et al, 2004; Kuznetsova et al, 2017). Public sequencing data are available for dragonfly (*Libellula Angelina* and *Ladona fulva*) (Consortium i5K, 2013), dainty damselfly (*Coenagrion scitulum*) (Consortium i5K, 2013), and mayfly (*Cloeon dipterum*) (Almudi et al, 2020). However, no predicted TRMs could be identified in any of these species using TRIP (Supplemental Data 1). We concluded that the ancestral TRM type (TTAGG)n is missing in Odonata and Ephemeroptera, which was an independent loss event compared with Dipterans (Fig 3). The mechanisms of chromosome end protection warrant further study in these orders.

Table S6 Telomeric Repeats Identification Pipeline candidate motif results for Dipteran species with retrotransposons or complex repeats as telomeres.

### Diverse Hymenoptera TRMs–(TTAGG)n is the ancestral TRM form in Hymenopterans

**Figure 4.**
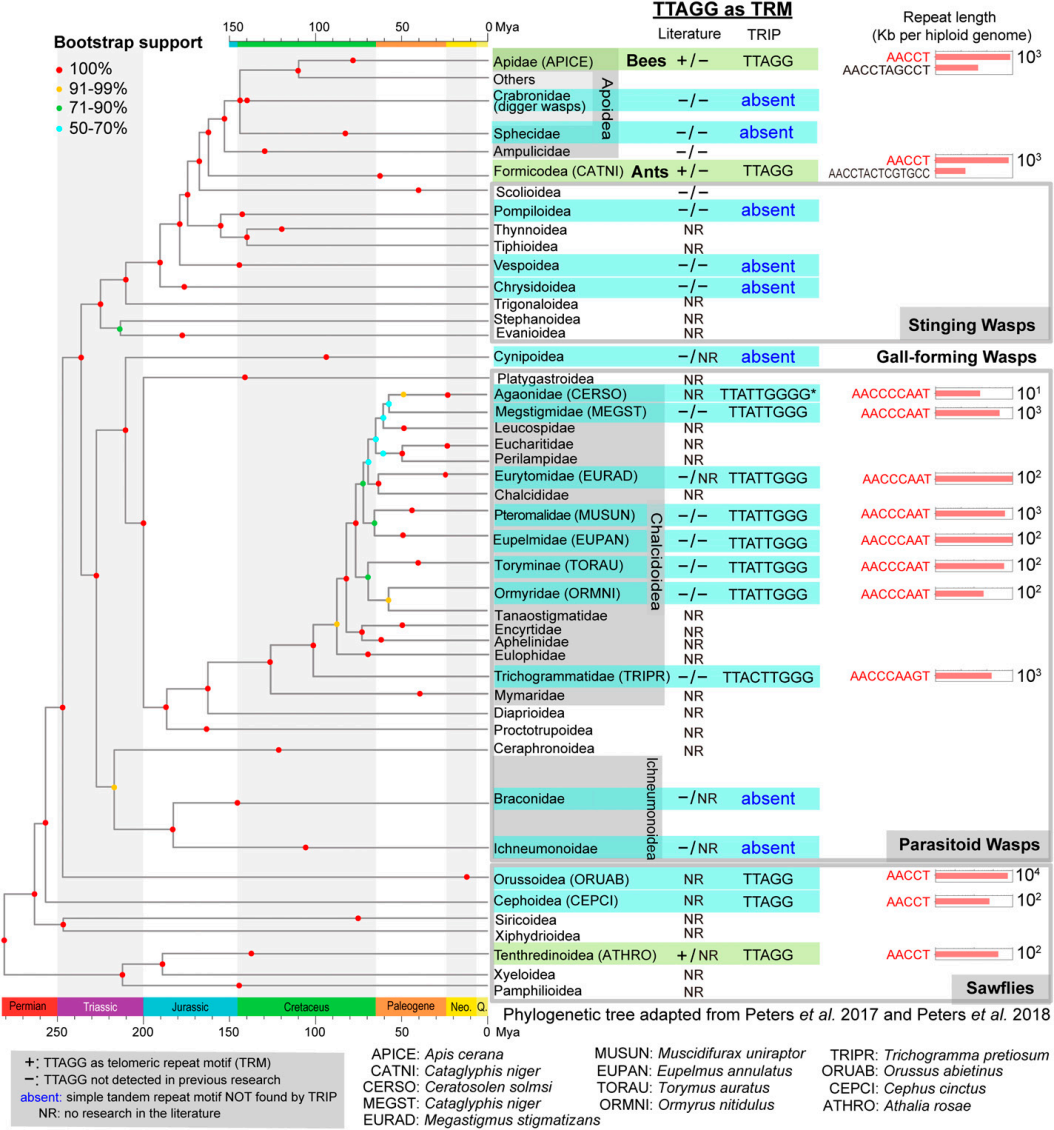
Profile of telomeric repeat motif (TRM) in Hymenopterans revealed elongated repeat monomers in parasitoid wasps. TRM identified from previous reports using (TTAGG)n probes are summarized in the middle panel next to the Hymenoptera phylogeny at the family level, adapted from Peters et al (2017, 2018). The “+” sign means confirmed (TTAGG)n TRM, and the “–” sign stands for lack of such signal. For Telomeric Repeats Identification Pipeline (TRIP) “+” families, the abundance (Kb per haploid genome) of candidate TRM are shown in horizontal bar plots for representative species. The branches are shaded in green if the TRIP results agree with previous literature report in any species within this taxon. The branches are shared in blue if novel TRM information is provided by TRIP.

(TTAGG)n was reported as the TRM in hymenopterans, and a longer 8-bp form was reported in a single wasp species *N. vitripennis* (Dalla Benetta et al, 2020), suggesting diverse forms of TRM in this order. To perform a comprehensive investigation of TRMs in Hymenoptera, we analyzed a total of 71 Hymenopteran species/strains in 14 superfamilies, and the results were shown on the Hymenopteran phylogeny (Fig 4). Sawflies are basal Hymenopterans, and the canonical insect TRM (TTAGG)n was found in two species in the superfamily Tenthredinidae using FISH (Gokhman & Kuznetsova, 2018). Genomic short-read data were available in three sawfly superfamilies, Tenthredinidae, Orussoidea, and Cephoidea, and TRIP identified (TTAGG)n as the predicted TRM in all of them (Fig 4 and Supplemental Data 4), providing strong evidence that TTAGG is the ancestral TRM in basal Hymenopterans. (TTAGG)n was also reported as the TRM form in advanced social insect clades, such as Apoidea (bees) and Formicoidea (ants). We confirmed the presence of (TTAGG)n as the TRM using TRIP (Fig 4 and Supplemental Data 4).

### Diverse Hymenoptera TRMs—elongated 8-bp TRMs in multiple parasitoid chalcid wasps with even longer motif variants in fig wasps and miniature wasps

Parasitoid wasps are the most abundant Hymenopteran and account for 20% of all insect species (Smith et al, 2008). Chalcidoidea is the largest Hymenopteran superfamily with more than half-million estimated species (Heraty, 2017). Among the 22 chalcid families, we generated/collected genome sequencing data for 10 families in 25 species/strains and found 8 or 9-bp TRMs in eight families (Fig 4 and Supplemental Data 4). The 8-bp (TTATTGGG)n was discovered to be the predicted TRM in Pteromalidae (*Nasonia*, *Trichomalopsis*, and *Muscidifurax*), Torymidae (*Torymus auratus*), Ormyridae (*Ormyrus pomaceus* and *Ormyrus nitidulus*), Megastigmidae (*Megastigmus stigmatizans* and *Bootanomyia dorsalis*), Eurytomidae (*Eurytoma adleriae*), and Eupelmidae (*Eupelmus annulatus*), suggesting the 8-bp unit is the dominant form in chalcid wasps (Fig 4). Interestingly, the basal family Trichogrammatidae, which are minute egg parasitoids with an adult body length of less than 1 mm (Lindsey et al, 2018), were discovered to have a 9-bp predicted TRM (TTACTTGGG)n by TRIP, with independent data support from two species *Trichomalopsis pretiosum* and *Trichogramma evanescens* (Fig 4 and Supplemental Data 4). We infer that this novel TRM originated from the 8-bp ancestral Chalcidoidea form by a single cytosine insertion in the repeat unit because the 9-bp repeat type is Trichogrammatidae specific.

Pollinating-fig wasps belong to the family Agaonidae. Their larvae feed inside the fig syconia, and pollinate the fig. This remarkable mutualism originated 66∼75 Mya (Heraty et al, 2013; Xiao et al, 2013, 2021; Peters et al, 2018). *Ceratosolen solmsi* is an important pollinator for its host tropical fig tree *Ficus hispida*, and we identified a 9-bp candidate TRM (TTATTGGGG)n, which differs from the 8-bp Chalcidoidea motif by a single Guanine addition (Fig 4 and Supplemental Data 4). Although this candidate TRM did not survive TRIP filtering, we observed at least four TRM regions at the very end of the assembled scaffolds (Xiao et al, 2013) (Table S7). This 9-bp TRM was also found at scaffold end in *Ceratosolen fusciceps*, another fig wasp species within this genus, providing independent evidence of this 9-bp novel TRM form (Table S7). To check whether this 9-bp TRM is common in all fig-pollinating wasps, we examined PacBio long-read assemblies of species from three additional fig wasp genera (*Kradibia, Dolichoris*, and *Eupristina*): *Kradibia gibbosae*, *Dolichoris vasculosae*, *Eupristina koningsbergeri* (Xiao et al, 2021), and *Eupristina verticillate* (Zhang et al, 2020). The 8-bp (TTATTGGG)n TRM was discovered at scaffold ends for all four species (Table S7), suggesting it is the ancestral form in Agaonidae. We conclude that the 8 to 9-bp TRM transition occurred in the common ancestor of *C. solmsi* and *C. fuscicep* after diverging from other Agaonidae genera. Based on a calibrated fig wasp phylogeny of 12 species (Xiao et al, 2021), we estimated this 9-bp TRM occurred between 55 and 43 Mya. Non-pollinating fig wasp species *Apocrypta bakeri* and *Sycobia* sp. (Xiao et al, 2021) in the family Pteromalidae have the 8-bp (TTATTGGG)n TRM (Table S8), which is the same form as other Pteromalidae species we examined. The *Ceratosolen*-specific TRM sequence turnover occurred within 55 million years after its divergence from *Kradibia*, showing a tendency of increasing TRM length in Chalcid wasps.

Table S7 Candidate telomeric regions in genome assemblies of pollinating fig wasps in the family of Agaonidae.

Table S8 Candidate telomeric regions in genome assemblies of non-pollinating fig wasps in the family of Pteromalidae.

### Diverse Hymenoptera TRMs–lack of TRM in Ichneumonoids, hunting wasps, and gall-forming wasps suggests multiple loss events during hymenopteran evolution

**Figure 5.**
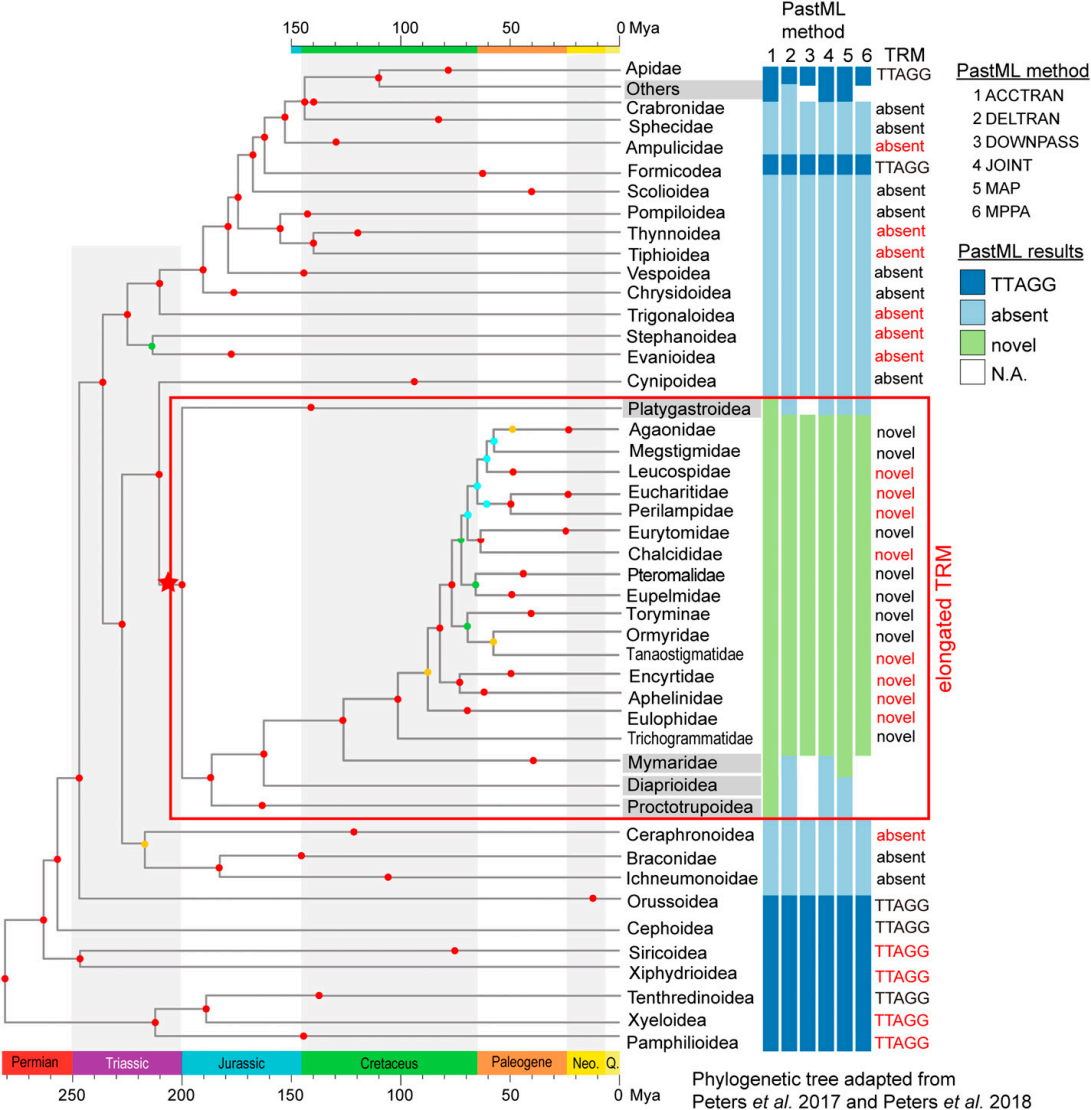
Ancestral telomeric repeat motif (TRM) states inference in Hymenopterans. Ancestral TRM states in Hymenopteran taxa inferred using PastML (Ishikawa et al, 2019). Three maximum parsimony (MP) methods (“1”: ACCTRAN; “2”: DELTRAN; “3”: DOWNPASS) and three maximum likelihood (ML) methods (4”: JOINT; “5”: MAP; “6”: MPPA) were used, and all six methods gave identical results for most taxa. Taxa with inconsistent state identification are shared in gray. The dark blue, light blue, green and white bars represents “TTAGG,” “absent,” “novel,” and “N.A. (not available)” TRM states, respectively. TRM with literature support and/or Telomeric Repeats Identification Pipeline calls are labeled in black, and unknown TRM states inferred by PastML are labeled in red. The red box includes taxa with inferred elongated TRM.

Despite the extensive sampling across Hymenoptera, we did not detect any predicted TRM in Ichneumonoids, hunting wasps, or gall-forming wasps. Ichneumonoidea is the second-largest Hymenopteran superfamily with ∼150,000 described species. In this study, nine species in the families Ichneumonidae and Braconidae were explored, and we did not identify any short tandem repeat form TRMs (Fig 4), which is consistent with the negative results from previous research using (TTAGG)n probes (Gokhman et al, 2014), suggesting the loss of ancestral (TTAGG)n telomeric repeats. Solitary hunting wasp families Crabronidae (digger wasps) and Sphecidae (sand wasp) lack TRM in our TRIP results, which are consistent with the negative signals in (TTAGG)n FISH experiments (Menezes et al, 2017). In stinging wasps, TRIP cannot identify any predicted TRMs in three superfamilies (Pompiloidea, Vespoidea, and Chrysidoidea). Together with the negative results of (TTAGG)n in Scolioidea (Menezes et al, 2017) in the literature, we hypothesized that a large-scale loss of simple tandem repeat type of TRM occurred in stinging wasp families (Fig 5 and Supplemental Data 4). Ten species in the gall-forming wasp family Cynipidae were also investigated, and no predicted TRMs were identified, suggesting a different mechanism of chromosome end protection in these herbivores.

### Diverse Hymenoptera TRMs—inferring TRMs by ancestral state reconstruction

A total of 22 families/superfamilies had no previous research about TRM in the literature, and they also lack genomic data for TRM identification by TRIP (Fig 4). To extend the knowledge of potential TRMs in these taxa, ancestral stae reconstruction was performed based on known TRM traits using six maximum parsimony and maximum likelihood methods implemented in PastML (Ishikawa et al, 2019). Among these 22 families/superfamilies, 19 had the same inferred TRM state from all six methods (Fig 5). Based on the ancestral TRM inference, basal sawflies (Pamphilioidea, Xyeloidea, Xiphydrioidea, and Siricoidea) have (TTAGG)n form TRM (Fig 5), and the five sting wasp taxa (Thynnoidea, Tiphioidea, Evanioidea, Trigonaloidea, and Stephanoidea) lack tandem repeat type TRMs (Fig 5). DELTRAN (delayed transformation) and DOWNPASS methods suggest that elongated TRM first appeared ∼100 Mya in Trichogrammatidae, whereas the MAP (maximum a posteriori) method supports the occurrence of ∼130 Mya in Mymaridae (Fig 5). Estimates from the ACCTRAN (accelerated transformation) method indicate an even more ancient origin ∼210 Mya in the ancestor of Platygastroidea (Fig 5). Additional experimental and genomic data are needed to resolve the exact evolutionary origin of the elongated TRMs. The current evidence suggests its origin between 100 and 210 Mya in parasitoid wasps.

### Characterization of TRM using TRIP and chromosome assembly revealed rapid evolution of TRM abundance in the parasitoid wasp family Pteromalidae

**Figure 6.**
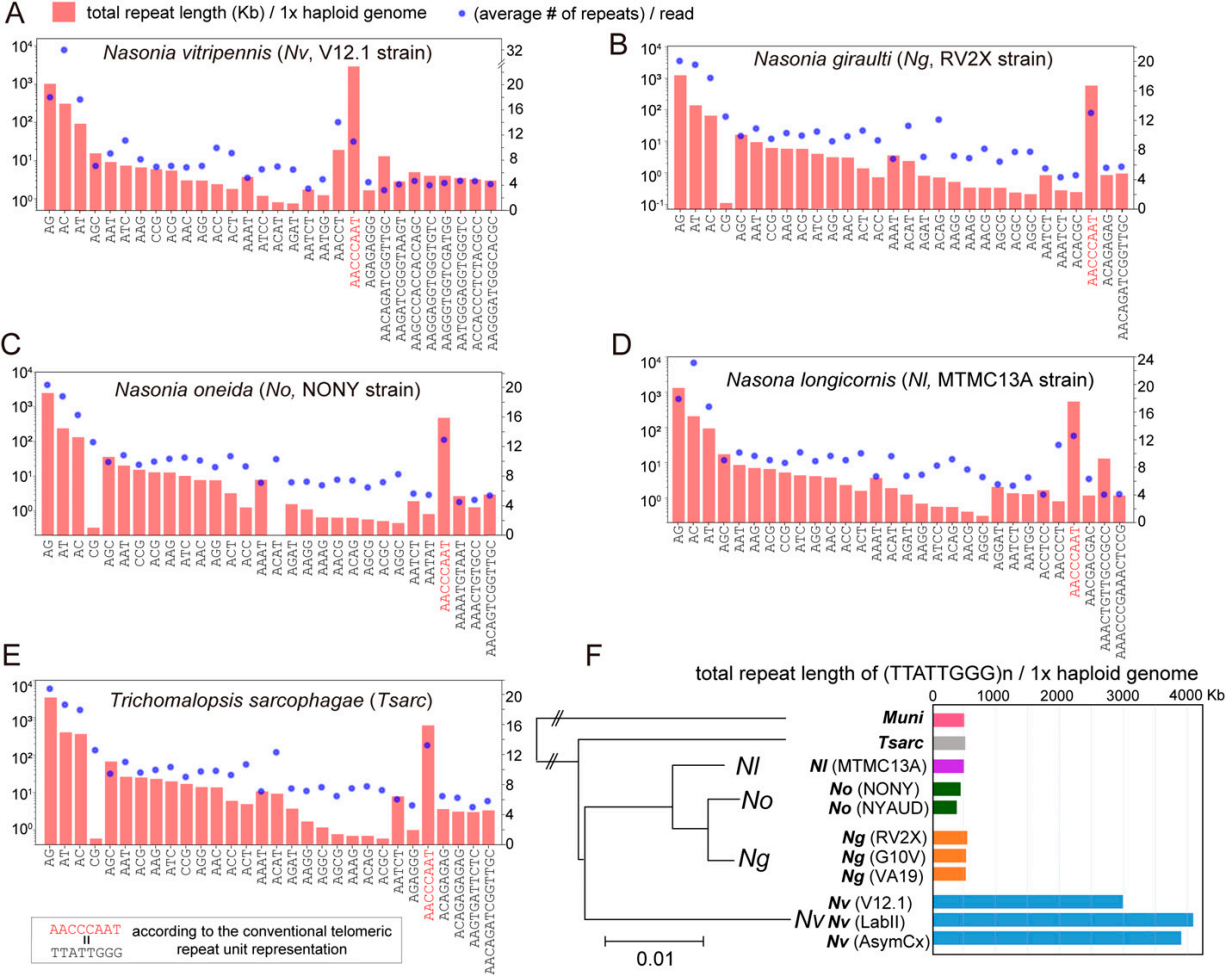
Rapid evolution of the (TTATTGGG)n telomeric repeat motif abundance in parasitoid wasps. **(A, B, C, D, E)** Top 30 simple tandem repeat motif profiles. **(A, B, C, D, E)** Total repeat length per haploid genome is plotted as a barplot in a log scale on the left *y*-axis, and the average number of repeats per read is plotted as a blue dot for each repeat motif (right *y*-axis), for *Nasonia vitripennis* (*Nv*) V12.1 strain (A), *Nasonia giraulti* (*Ng*) RV2X strain (B), *Nasonia oneida* (*No*) NONY strain (C), *Nasonia longicornis* (*Nl*) MTMC13A strain (D), and *Trichomalopsis sarcophagae* (*Tsarc*) (E). **(F)** A horizontal bar plot of total telomeric repeat motif length per haploid genome in 11 parasitoid wasp species/strains in the family Pteromalidae. *Muni*, *Muscidifurax uniraptor*.

To confirm that the (TTATTGGG)n TRM is the common form rather than a specific case in *Nv*, we performed Illumina short-read sequencing of the genomes of 11 wasp strains in six species of the family Pteromalidae, with an average coverage of 295× (see the Materials and Methods section). (TTATTGGG)n was identified as the predicted TRM in all of them (Fig 6A–E). In addition, we performed PacBio long-read sequencing of four parasitoid wasp species (Xiong et al, 2021) and achieved chromosome-level assemblies (Table S9). These wasps have a haploid karyotype of *n* = 5 (Goodpasture, 1974; Gokhman & Westendorff, 2000; Silva-Junior et al, 2000). (TTATTGGG)n was found at all 10 chromosome termini in all four species (Table S9), serving as a definitive validation of the TRM calls. We also quantified the total telomeric repeat length per haploid genome and discovered that the three *Nv* strains (AsymCx, LabII, and V12.1) have an average length of 3,856 Kbp, a 6.5-fold increase in abundance compared with five other related wasp species (*P* = 0.019, Mann–Whitney U test), despite having the same chromosome number (Fig 6F). *Trichomalopsis*, *Muscidifurax*, and *Nasonia* only diverged within 4.9 Mya, suggesting that rapid TRM expansion occurred in *Nv* within 1 million years since it diverged from its close relative *Nasonia giraulti* (*Ng*).

Table S9 Telomeric repeat motif calls for four parasitoid wasps species from chromosome-level genome assembly.

## Discussion

### A fast and accurate pipeline for TRM discovery from short-read genome sequencing data

We developed a TRIP to identify predicted TRM by using massive publicly available short-read sequencing data with the following advantages. It makes use of existing WGS short-read data, whereas the hybridization and FISH approaches require prior knowledge or speculation about the TRM for probe design, which is not capable of identifying novel TRMs or confirming the absence of TRM. The TRIP pipeline can perform de novo TRM detection, and negative results suggest a lack of simple tandem repeat–type telomere. High-quality chromosome-level assembly from a combination of long-read sequencing, Hi-C, and optical mapping can be used for direct identification of TRM at chromosome ends. Still, it is currently cost-prohibitive to use for all species. Not all chromosomes were assembled with intact termini because chromosome ends are more likely to be damaged during DNA extraction and fragmentation. As short-read data become available for more species, the TRIP pipeline will serve as a standard genome analysis tool for predicted TRM identification and provide strong candidate motifs for other experimental methods. Although long-read sequencing and genome assembly were performed in selected species to support the terminal location of these identified TRMs, further experimental analysis of the chromosome termini location at the cytogenetic level has yet to be performed.

### The effect of interstitial telomere-like sequences on TRIP TRM identification

TRIP is a short tandem repeat–based approach, so it is subject to the issue of spurious calls of interstitial telomeric repeat–like sequences. Identification of highly abundant telomere-like tandem repeats does not mean they serve as telomeric repeats. In this study, we tested TRIP in 91 species spanning nine classes and did not observe any false-positive cases. Although we cannot completely exclude this possibility, interstitial telomeric repeat–like sequences are extremely unlikely to affect TRIP results. This is because we only allow 100% match in tandem motif identification in the sequencing reads. Interstitial telomeric repeats were not known to be under selective pressure so they could mutate freely. Most sequencing reads containing these interstitial telomeric repeat–like were excluded from the repeat counts because of the non-perfect match in the motif sequences. If noncanonical TRMs were identified, experimental confirmation using the Bal31 digestion or FISH approaches, as well as direct identification from chromosome-level assemblies would be needed to validate the findings.

### Ancestral (TTAGG)n TRM is conserved in most insect orders

Our extensive profiling of TRMs in major insect orders provided additional evidence to confirm that (TTAGG)n is the ancestral TRM in Insecta, as reported and summarized in the literature (Sahara et al, 1999; Sasaki & Fujiwara, 2000; Lorite et al, 2002; Frydrychova et al, 2004; Vitkova et al, 2005; Korandova et al, 2014; Menezes et al, 2017; Kuznetsova et al, 2019; de Castro et al, 2020). In addition to the insect orders, we discovered the (TTAGG)n TRM in Diplura, which is the immediate outgroup of Insecta (Fig 3). Nineteen of 25 informative Insecta orders have the (TTAGG)n motif, which confirms (TTAGG)n as the ancestral Insecta TRM, through this largest-scale survey across Insecta to date.

### Rapid evolving elongated TRM in parasitoid wasps indicates intriguing evolutionary fluidity of the Hymenopteran TRM

TRM sequences are highly conserved. In addition to the multiple insect orders we discussed, ancestral TRM (TTAGG)n was also identified in Chelicerata, Myriapoda, Protura, and Diplura, suggesting that it remained unchanged for more than 550 million years, before the Cambrian explosion. In deuterostomes, (TTAGGG)n was found to be the TRM in all species investigated so far (Jain & Cooper, 2010; Kondo & Akasaka, 2012; Bliznina et al, 2021), which diverged ∼600 Mya (Delsuc et al, 2018). In contrast, the telomere repeat motif in parasitoid wasps evolved from (TTAGG)n to a long repeat pattern (TTATTGGG)n, which occurred 110∼220 Mya. An even longer TRM, (TTACTTGGG)n, was identified in Trichogrammatidae, by adding a C in the AT-rich region, which was presumably occurred ∼100 Mya (Fig 4). As the common form in parasitoid wasps, (TTATTGGG)n was also found in *E. verticillate*, a pollinating-fig wasp species in the family Agaonidae. Another 9-bp variant (TTATTGGGG)n was identified in *Ceratosolen*, which is an example of novel TRM in the same family within 55 million years. Compared to the unaltered insect and deuterostome ancestral TRMs (TTAGG and TTAGGG), the TRM sequence turnovers we identified in parasitoid Chalcid wasps are extremely recent, suggesting rapid evolution of telomere sequences.

### Concerted sequence changes in the TERC template domain and telomere-binding proteins in parasitoid wasps?

Telomerase is responsible for restoring the telomere length after each cell division. As a reverse transcriptase, telomerase is a ribonucleoprotein using its RNA component (TERC) as a template to elongate the telomere. TERC RNA lacks sequence conservation even among closely related species, and the functional conservation is presumably achieved through secondary structures (Logeswaran et al, 2021). The repeat template regions in the TERC RNA have 100% sequence identity to the TRM. However, changes in TRM sequence during evolution may not require alterations in the conserved TERC template region. For example, the major forms of insect (TTAGG) and deuterostome (TTAGGG) TRMs are compatible with the TTA(G)n pattern, which is TTA followed by adjacent Gs. They could be synthesized from the same TERC core template TTAGGG (Fig 1). Surprisingly, the newly discovered elongated TRMs in parasitoid wasps (TTATTGGG and others) are not compatible with the ancestral TTA(G)n pattern. Therefore, sequence evolution in the TERC template must have occurred, unless a unique and complicated template shift mechanism is responsible for the synthesis of the longer TRM variants. The fig wasp TRM (TTATTGGGG) may share the same TERC template as TTATTGGG containing species, but the 9-bp Trichogrammatidae TRM contains an extra C in the middle of the unit (TTACTTGGG), and an insertion of a C is expected to occur in the 8-bp ancestral Chalcidoidea TERC template region. In addition to TERC evolution, there are more than 500 telomere-interacting proteins in human and mouse, many of which directly bind the (TTAGGG)n sequences (Shore, 1997; Luo et al, 2015; Braun et al, 2018). The rapid evolution of TRM in chalcid wasps may cause concerted evolution in the binding sites of telomere-binding proteins, to maintain the critical function of telomere elongation and regulation. Characterizing the TERC RNA, telomerase, and telomere-binding proteins in Hymenopterans will allow researchers to test these hypotheses formally and elucidate telomere-associated RNA and protein evolution.

### New insights on the canonical TRM pattern

The canonical TRM discovered in previous studies could be summarized as the formula T_x_A_y_G_z_ (TTAGG, TTAGGG, TTTAGGG, etc.) (Peska & Garcia, 2020). There are a few exceptions, but the outliers were not commonly found in a monophyletic group. Our discoveries shed new light on the TRM formula, and the common pattern is refined as W_a_C_b_W_c_G_d_ (W: A/T, b = 0 or 1, d = 2–4), which consists of an A/T region followed by a G-overhang with 2–4 consecutive Gs. At most, a single C insertion can be tolerated in the A/T region to accommodate the (TCAGG)n TRM found in Coleoptera, and the (TTACTTGGG)n motif in *Trichogramma* discovered in this research. This formula is also consistent with the TRM discovered in yeast (Wellinger & Zakian, 2012) and green algae (Fulneckova et al, 2012). However, in Saccharomycetaceae yeast, a large number of diverse long forms of G-quadruplexes related TRM were found through tandem repeat predictions (Cervenak et al, 2019; Peska et al, 2021). The TTATGGGCTCGG telomeric repeat discovered in Allium plants is another long-form TRM (Fajkus et al, 2016). These outliers were not consistent with the formula we proposed.

### Origin of elongated and variable TRMs in parasitoid wasps, and diverse forms of telomeric repeats in Hymenopterans

In Hymenopterans, the canonical 5-bp (TTAGG)n is likely to be the ancestral form TRM because it is identified in all basal sawfly taxa. At least three independent TRM loss events occurred in Ichneumonoids, hunting wasps, and gall-forming wasps at ∼210 Mya. Surprisingly, we discovered an 8-bp (TTATTGGG)n TRM common in parasitoid species, which is 60% longer than known Metazoan motifs. The 8-bp or longer TRM forms were only present in Chalcidoidea, which is a superfamily of parasitoid wasps that experienced massive radiation ∼200 Mya (Peters et al, 2017). It is a mystery why rapid sequence evolution occurred on the highly conserved telomeric repeats with essential functions to protect the chromosome termini. In addition, total TRM repeat length expansion was observed within the genus and species level (Fig 6), indicating active evolution within 1 million years. The evolutionary origin and sequence turnover of these long-form TRMs warrant further study.

## Materials and Methods

### The TRIP workflow

TRIP was designed for the identification of candidate TRMs using automated tandem repeats abundance profiling from short-read sequencing data 75–250 bp in length. With provided species information table, TRIP can automatically create a local repository, fetch data from European Nucleotide Archive and NCBI, process the short-read data, generate summary tables, visualize the repeats distribution, and return the final TRM identification results. The read processing step is achieved by an ultra-fast tandem repeat detector RepeatMaster. RepeatMaster is composed of RepeatDetector and RepeatSummary. The WGS read data in FASTQ or compressed format will be processed by RepeatDetector to output an intermediate table of all short tandem repeats with unit size ranging from 2 to 25 bp. Subsequently, RepeatSummary will summarize and quantify the repeat abundance in a tab-delimited table for downstream TRIP analysis. RepeatMaster is offered as independent software, which enables TRIP to handle local data in addition to publicly available data. TRIP will compute 19 parameters (Table S10) from RepeatMaster output, five of which are used to characterize and identify the TRM candidates (Table S1). A set of two filtering criteria is applied to make the final TRM call (Table S2). The sequencing processing speed is ∼10 Kbp per second per process with negligible memory cost. The tested working environment is Linux 2.6.32 using Intel Xeon Processor E5-2660 CPU at 2.20 GHz. TRIP is open-source under GPL-3.0 License.

Table S10 Tandem repeat statistics computed in Telomeric Repeats Identification Pipeline.

Supplemental Data 1. The list of TRIP telomeric repeat motif (TRM) calls and data sources for 91 species with known TRM in the literature.

Supplemental Data 2. Tandem repeat statistics and TRIP TRM calls for 41 species with simple tandem type TRM.

Supplemental Data 3. Tandem repeat statistics for 50 species without simple tandem type TRM.

Supplemental Data 4. TRM identification results and genome data statistics for insect species covered in this research.

Supplemental Data 5. Tandem repeat statistics and TRIP TRM calls for arthropods species that are not listed in Supplemental Data 2 or Supplemental Data 3.

Supplemental Data 6. Candidate telomeric repeat motifs identified in this research.

Supplemental Data 7. The listed of data source and accession numbers for all genome datasets used in this research.

### Data acquisition from publicly available databases

Short-read WGS data used in this research were downloaded from NCBI (NCBI Resource Coordinators, 2018), Sequence Read Archive (Leinonen et al, 2011), and European Nucleotide Archive (Harrison et al, 2021). We selected sequencing datasets with at least 10× haploid genome coverage (can be specified using --avg_genome_cov parameter in TRIP), preferably with a reference genome assembly. The accession number for all datasets used were listed in Supplemental Data 7. For direct identification and confirmation of predicted TRMs, long-read PacBio assemblies of wasps in the families Agaonidae and Pteromalidae examined in this studies were downloaded from NCBI Assembly with accession numbers https://www.ncbi.nlm.nih.gov/assembly/GCA_009193385.2/, https://www.ncbi.nlm.nih.gov/assembly/GCA_020010945.1/, https://www.ncbi.nlm.nih.gov/assembly/GCA_018907195.1/, https://www.ncbi.nlm.nih.gov/assembly/GCA_018907245.1/, https://www.ncbi.nlm.nih.gov/assembly/GCA_000503995.1/, https://www.ncbi.nlm.nih.gov/assembly/GCA_018883505.1/, https://www.ncbi.nlm.nih.gov/assembly/GCA_018907135.1/, https://www.ncbi.nlm.nih.gov/assembly/GCA_018906985.1/, https://www.ncbi.nlm.nih.gov/assembly/GCA_018907035.1/, and National Genomics Data Center (http://ngdc.cncb.ac.cn) with accession number https://ngdc.cncb.ac.cn/search/?dbId=&q=GWHALOE00000000.

### WGS and genome assembly of the silverfish *L. saccharina*

*L. saccharina* was collected from a colony maintained at Auburn University in the laboratory environment. DNA sample was extracted from a single adult male using QIAGEN MagAttract HMW DNA Mini Kit (QIAGEN). DNA concentration was measured on a Qubit 3.0 Fluorometer (Thermo Fisher Scientific). The quality and size distribution were accessed on Agilent TapeStation 4200 (Agilent Technologies) using Agilent Genomic DNA ScreenTape Assay. The DNA integrity number (DIN) is 9.3, suggesting perfect DNA integrity. A 10× Genomic library was constructed using the Chromium Genome Reagent Kits v2 on the Chromium Controller (10x Genomics Inc.) with 1.2 ng input DNA. After quality control, the library was sequenced on an Illumina HiSeq X machine at the Genomic Services Lab at the HudsonAlpha Institute for Biotechnology.

A total of 895 million 150-bp reads were obtained (134.2 Gbp of sequences). An initial de novo assembly of *L. saccharina* genome was performed by the Supernova assembler version v2.1.1 (Weisenfeld et al, 2017) using linked reads information. To improve the assembly, we carried out an additional de novo assembly using MEGAHIT version 1.2.9 (Li et al, 2015), using high-quality reads trimmed by Trimmomatic v0.39 (Bolger et al, 2014). These two assemblies were combined using quickmerge (Chakraborty et al, 2016). Scaffolds with a size greater than 2 Kb were kept in the final assembly.

### Genome sequencing and assembly of parasitoid wasp species

The genomes of 11 strains of parasitoid wasps were sequenced in our previous research (Wang et al, 2020; Lin et al, 2021; Xiong et al, 2021). These strains are highly inbred and are maintained in our laboratory under constant lighting conditions at 25°C. *N. vitripennis* reference strain (AsymCx), a laboratory strain derived from isolates in the Netherlands (LabII), and a laboratory strain originated from Rochester, New York (V12.1), were sequenced using 10× Genomics approach. Six strains from three closely related species in the *Nasonia* genus were also included: *N. giraulti* RV2X strain, *N. giraulti* G10 strain with *Ng* nuclear genome and *Nv* mitochondrial genome background, *N. giraulti* V19_008U strain collected in Virginia, *Nasonia oneida* NONY 11/36 strain, *N. oneida* NONYAUD108 strain, and *Nasonia longicornis* MTMC13A strain. Two species outside the *Nasonia* genus, *Trichomalopsis sarcophagae*, and *Muscidifurax uniratpor*, were selected for DNA extraction and genome sequencing.

### Reconstruction of ancestral TRM states in Hymenopterans

Construction of ancestral TRM states was performed using PastML (Ishikawa et al, 2019) with six methods, including three maximum parsimony ACCTRAN (accelerated transformation), DELTRAN (delayed transformation), and DOWNPASS, as well as three maximum likelihood methods JOINT, MAP (maximum a posteriori), and MPPA (marginal posterior probabilities approximation). The Newick tree file and TRM traits (TTAGG, absent, and novel) were provided as input.

## Data Availability

The TRIP pipeline can be downloaded at https://github.com/XuWangLab/TRIP; http://github.com/XuWangLab/2020_insectTelomere_sppData. The genome assembly of *L. saccharina* can be accessed at NCBI using accession number https://www.ncbi.nlm.nih.gov/nuccore/JAGDQP000000000, and the raw sequencing data have been deposited to NCBI database and assigned the identifier https://www.ncbi.nlm.nih.gov/bioproject/?term=PRJNA707018.

## Supplementary Material

Reviewer comments
