# Supplementary material for: Profiles of telomeric repeats in Insecta reveal diverse forms of telomeric motifs in Hymenopterans
Source: Life Sci Alliance. 2022 Apr 1;5(7):e202101163. doi: 10.26508/lsa.202101163 (PMC8977481; doi:10.26508/lsa.202101163)
Supplement: Supplementary file 1 [file LSA-2021-01163_TableS1.docx]

**Table S1.** **Telomeric repeat motif candidates identification in TRIP.**

| **Parameters for TRM candidates*** | **Criteria** | **Purpose** |
| --- | --- | --- |
| (1) avg_genome_cov  (2) rpt_reads_num | ≥ 10x  ≥ 12,000 | Ensure sufficient amount of input data  Make sure the simple repeats were not pre-filtered in the raw data |
| (3) repeats_len_per_million_reads | ≥ 4 Kb | Meet a minimum total repeat length requirement (telomeres consist of hundreds to thousands base pairs). |
| (4) percent_repeats_len_per_read | ≥ 50% | Exclude intersperse short-tandem repeats (telomeres are tandem repeats with a large number of copies). |
| (5) unit_len | ≥ 5 bp | Meet a minimum of TRM unit length requirement (all known telomere motifs are 5 bp or longer). |

* See Table S1 for definitions.
