# Supplementary material for: Profiles of telomeric repeats in Insecta reveal diverse forms of telomeric motifs in Hymenopterans
Source: Life Sci Alliance. 2022 Apr 1;5(7):e202101163. doi: 10.26508/lsa.202101163 (PMC8977481; doi:10.26508/lsa.202101163)
Supplement: Supplementary file 2 [file LSA-2021-01163_TableS2.docx]

**Table S2. Telomeric repeat motif calling criteria in TRIP.**

| **Criteria for TRM call** | **Purpose** |
| --- | --- |
| (1) best_candidate_enrichment* ≥ 3 fold | **the “dominant” rule** to ensure a single best hit (only one TRM call for any given species) |
| (2) the same pattern is observed in the same family from the literature or TRIP results | **the “support” rule** to eliminate false positives (TRM are highly conserved which are unlikely to differ with family) |
| OR  TRM observed directly from genome assembly | If ≥ 40bp TR motif regions were observed at the very end of ≥ 4 chromosomes/scaffolds in a high-quality genome assembly (scaffold N50 ≥ 1Mb), the support rule can be overridden. |

* See Table S1 for definitions.
