# Supplementary material for: Profiles of telomeric repeats in Insecta reveal diverse forms of telomeric motifs in Hymenopterans
Source: Life Sci Alliance. 2022 Apr 1;5(7):e202101163. doi: 10.26508/lsa.202101163 (PMC8977481; doi:10.26508/lsa.202101163)
Supplement: Supplementary file 3 [file LSA-2021-01163_TableS3.docx]

**Table S3. *Nasonia vitripennis* genome mapping results of TTATTGGG neighboring sub-telomeric region assembly.**

| **Assembled subtelomeric contig** | **Scaffold alignment  coordinates*** | **% sequence identity** | **BLAT E-value** | **Chr mapping results** |
| --- | --- | --- | --- | --- |
| k141_435 | SCAFFOLD16: 1-102 | 100% | 2.87E-46 | chr1_start |
| k141_288 | SCAFFOLD116: 738598-738720 | 95.12% | 4.38E-48 | chr2_start |
| k141_435 | SCAFFOLD309: 3-403 | 86.63% | 3.36E-115 | chr2_end |
| k141_273 | SCAFFOLD18: 3693741-3693482 | 88.46% | 1.28E-84 | chr3_start |
| k141_435 | SCAFFOLD28: 1899952-1899551 | 96.77% | 0 | chr3_end |
| k141_321 | SCAFFOLD4: 5241320-5241887 | 100% | 0 | chr4_start |
| k141_395 | SCAFFOLD9: 4815-4930 | 100% | 2.74E-54 | chr4_end |
| k141_435 | SCAFFOLD2: 1-307 | 96.10% | 3.25E-140 | chr5_end |

* Physical location from *Nasonia vitripennis* reference assembly v2.1 (GCA_000002325.2).
