# Supplementary material for: Profiles of telomeric repeats in Insecta reveal diverse forms of telomeric motifs in Hymenopterans
Source: Life Sci Alliance. 2022 Apr 1;5(7):e202101163. doi: 10.26508/lsa.202101163 (PMC8977481; doi:10.26508/lsa.202101163)
Supplement: Supplementary file 4 [file LSA-2021-01163_TableS4.docx]

**Table S4. Telomeric length and number telomeric repeat motifs in the *Nv* PSR genome assembly.**

| **Chromosome*** | **Chr length (bp)** | **TRM coordinates** | **TRM motif** | **TRM length (bp)** |
| --- | --- | --- | --- | --- |
| chr1 | 37,874,440 | chr1: 1-4,432 | TTATTGGG | 4432 |
| chr1 | 37,874,440 | chr1: 37,872,035-37,874,440 | TTATTGGG | 2406 |
| chr2 | 35,393,729 | chr2: 1-3,697 | TTATTGGG | 3697 |
| chr2 | 35,393,729 | chr2: 35,389,332-35,393,729 | TTATTGGG | 4398 |
| chr3 | 24,760,113 | chr3: 24,758,365-24,760,113 | TTATTGGG | 1749 |
| chr4 | 33,170,505 | chr4: 1-3,029 | TTATTGGG | 3029 |
| chr5 | 28,269,460 | chr5: 28,268,130-28,269,460 | TTATTGGG | 1331 |

* Chromosome assembly and physical locations from *Nasonia vitripennis* PSR strain assembly v1.1 (GCA_009193385.2).
