# Supplementary material for: Profiles of telomeric repeats in Insecta reveal diverse forms of telomeric motifs in Hymenopterans
Source: Life Sci Alliance. 2022 Apr 1;5(7):e202101163. doi: 10.26508/lsa.202101163 (PMC8977481; doi:10.26508/lsa.202101163)
Supplement: Supplementary file 5 [file LSA-2021-01163_TableS5.docx]

**Table S5. Major Insecta order and Hymenopteran superfamily with no or insufficient sequencing data for telomeric repeat motif call.**

| **Insecta order** | **data  availability** | **Sequencing  depth** | **Assembly  availability** | **Observed in assembly** |
| --- | --- | --- | --- | --- |
| Dermaptera | NO | N/A | NO | NO |
| Grylloblattodea | NO | N/A | NO | NO |
| Embioptera | NO | N/A | NO | NO |
| Raphidioptera | NO | N/A | NO | NO |
| Siphonaptera | YES | 25×* | YES | NO |
| Mecoptera | YES | < 10× | NO | NO |
| Megaloptera | YES | < 10× | YES | NO |
| Grylloblattodea | NO | N/A | NO | NO |
| Mantophasmatodea | NO | N/A | NO | NO |
| Zoraptera | NO | N/A | NO | NO |
| **Hymenopteran Superfamily** |  |  |  |  |
| Scoliodea | YES | < 10× | NO | NO |
| Thynnoidea | NO | N/A | NO | NO |
| Tiphioidea | NO | N/A | NO | NO |
| Trigonaloidea | NO | < 10× | NO | NO |
| Stephanoidea | NO | N/A | NO | NO |
| Platygastroidea | YES | < 10× | NO | NO |
| Diaprioidea | YES | < 10× | NO | NO |
| Proctotrupoidea | YES | < 10× | NO | NO |
| Ceraphronoidea | NO | N/A | NO | NO |
| Siricoidea | NO | N/A | NO | NO |
| Xiphydrioidea | NO | N/A | NO | NO |
| Pamphilioidea | NO | N/A | NO | NO |

* raw data not provided by authors.
