# Supplementary material for: Profiles of telomeric repeats in Insecta reveal diverse forms of telomeric motifs in Hymenopterans
Source: Life Sci Alliance. 2022 Apr 1;5(7):e202101163. doi: 10.26508/lsa.202101163 (PMC8977481; doi:10.26508/lsa.202101163)
Supplement: Supplementary file 6 [file LSA-2021-01163_TableS6.docx]

**Table S6. TRIP candidate motif results for Dipteran species with retrotransposons or complex repeats as telomeres.**

| ***Drosophila melanogaster*** | **Reads number** | **Repeat number** | **Repeat length** |
| --- | --- | --- | --- |
| AAGAG | 6,295,175 | 96,606,062 | 483,030,310 |
| AACATAGAAT | 2,410,968 | 14,835,560 | 148,355,600 |
| AACAC | 938,079 | 16,202,933 | 81,014,665 |
| AAGAC | 728,058 | 11,148,482 | 55,742,410 |
| AAACAC | 203,630 | 2,891,793 | 17,350,758 |
| AAGAGAG | 198,888 | 1,749,390 | 12,245,730 |
| AAACACAACAC | 68,852 | 531,611 | 5,847,721 |
| AAGAT | 93,995 | 1,121,966 | 5,609,830 |
| AAAAG | 75,871 | 1,061,990 | 5,309,950 |
| AAGGC | 81,089 | 1,027,492 | 5,137,460 |
| AAGGAG | 51,503 | 765,474 | 4,592,844 |
| AACATAT | 37,506 | 518,080 | 3,626,560 |
| AATAG | 32,538 | 516,960 | 2,584,800 |
| ***Aedes albopictus*** | **Reads number** | **Repeat number** | **Repeat length** |
| AAATCCAATCC | 381,458 | 2,842,810 | 31,270,910 |
| AAGATAG | 141,344 | 1,956,346 | 13,694,422 |
| AAGACAGAG | 112,239 | 1,077,426 | 9,696,834 |
| AAATCGAATCG | 73,861 | 615,611 | 6,771,721 |
| ACTAG | 72,338 | 1,187,906 | 5,939,530 |
| AAGAAGAC | 56,649 | 707,319 | 5,658,552 |
| AAAGC | 58,911 | 1,093,224 | 5,466,120 |
| AAAATTAAATT | 49,386 | 452,425 | 4,976,675 |
| AAGACTAG | 53,766 | 494,608 | 3,956,864 |
| AAAAGGTCTTTAGGCCG | 43,465 | 229,658 | 3,904,186 |
| AAAATT | 32,284 | 540,216 | 3,241,296 |
| AAGACCAG | 42,529 | 350,423 | 2,803,384 |
| AGAGC | 30,449 | 545,305 | 2,726,525 |
| AACAGACAG | 30,870 | 283,013 | 2,547,117 |
| ACTCT | 26,517 | 507,102 | 2,535,510 |
| AAACAAAT | 28,340 | 314,588 | 2,516,704 |
| AGAGATGAGATG | 33,598 | 188,373 | 2,260,476 |
| AGAGATG | 27,718 | 314,309 | 2,200,163 |
| AAGACAG | 22,552 | 297,278 | 2,080,946 |
| AAGGACTTCCTGGAGGAATCCCCG | 18,600 | 76,403 | 1,833,672 |
