# Supplementary material for: Profiles of telomeric repeats in Insecta reveal diverse forms of telomeric motifs in Hymenopterans
Source: Life Sci Alliance. 2022 Apr 1;5(7):e202101163. doi: 10.26508/lsa.202101163 (PMC8977481; doi:10.26508/lsa.202101163)
Supplement: Supplementary file 7 [file LSA-2021-01163_TableS7.docx]

**Table S7. Candidate telomeri regions in genome assemblies of pollenating fig wasps in the family of Agaonidae.**

| **Species** | **Scaffold** | **Scaffold length (bp)** | **Candidate TRM region** | **TRM unit** | **TRM length in scaffold (bp)** |
| --- | --- | --- | --- | --- | --- |
| *Ceratosolen solmsi* | NW_011948416.1 | 15,084,686 | 15,084,645-15,084,686 | TTATTGGGG | 42 |
|  | NW_011948439.1 | 2,341,666 | 1-41 | TTATTGGGG | 41 |
|  | NW_011948436.1 | 3,995,362 | 1-42 | TTATTGGGG | 42 |
|  | NW_011948479.1 | 26,146 | 1-40 | TTATTGGGG | 40 |
| *Ceratosolen fusciceps* | RCIC01000045.1 | 887,815 | 887,525-887,754 | TTATTGGGG | 230 |
| *Kradibia gibbosae* | JACCHX010000005.1 | 21,929,900 | 1-3,672 | TTATTGGG | 3,672 |
|  | JACCHX010000010.1 | 6,817,775 | 1-3,565 | TTATTGGG | 3,565 |
| *Dolichoris vasculosae* | JACCHY010000001.1 | 30,650,846 | 451-849 | TTATTGGG | 399 |
|  | JACCHY010000002.1 | 30,042,859 | 27,039,949-27,041,977 | TTATTGGG | 2,029 |
|  | JACCHY010000008.1 | 10,894,738 | 10,893,407-10,894,558 | TTATTGGG | 1,152 |
|  | JACCHY010000009.1 | 8,252,283 | 8,248,102-8,251,402 | TTATTGGG | 3,301 |
|  | JACCHY010000021.1 | 4,587,590 | 4,587,454-4,587,590 | TTATTGGG | 137 |
|  | JACCHY010000022.1 | 4,227,587 | 1-535 | TTATTGGG | 535 |
|  | JACCHY010000030.1 | 2,879,229 | 329-2,867 | TTATTGGG | 2,539 |
|  | JACCHY010000035.1* | 1,610,072 | 1,607,644-1,609,451 | TTATTGGG | 1,808 |
| *Eupristina verticillate* | GWHALOE00000159 | 2,272,118 | 2,271,449-2,272,118 | TTATTGGG | 670 |
|  | GWHALOE00000761 | 6,827,271 | 6,826,551-6,827,271 | TTATTGGG | 720 |
|  | GWHALOE00000232 | 996,484 | 1-143 | TTATTGGG | 143 |
|  | GWHALOE00000339** | 1,867,337 | 1-553 | TTATTGGG | 553 |
|  | GWHALOE00000468 | 1,135,673 | 1-157 | TTATTGGG | 157 |
| *Eupristina koningsbergeri* | JACCHV010000165.1 | 396,311 | 395,907-396,311 | TTATTGGG | 405 |
|  | JACCHV010000203.1 | 54,390 | 1-200 | TTATTGGG | 200 |
|  | JACCHV010000065.1 | 21,044 | 1-340 | TTATTGGG | 340 |
|  | JACCHV010000416.1 | 14,279 | 1-277 | TTATTGGG | 277 |
|  | JACCHV010000955.1 | 5,954 | 1-121 | TTATTGGG | 121 |

* 161 bp T-rich simple sequence insertion.

** 1bp deletion found in 22.8% repeat monomers.
