# Supplementary material for: Profiles of telomeric repeats in Insecta reveal diverse forms of telomeric motifs in Hymenopterans
Source: Life Sci Alliance. 2022 Apr 1;5(7):e202101163. doi: 10.26508/lsa.202101163 (PMC8977481; doi:10.26508/lsa.202101163)
Supplement: Supplementary file 8 [file LSA-2021-01163_TableS8.docx]

**Table S8. Candidate telomeri regions in genome assemblies of non-pollenating fig wasps in the family of Pteromalidae.**

| **Species** | **Scaffold** | **Scaffold size (bp)** | **Candidate TRM region** | **TRM unit** | **TRM total length (bp)** |
| --- | --- | --- | --- | --- | --- |
| *Apocrypta bakeri* | JACCIA010000002.1 | 25,026,353 | 1-93 | TTATTGGG | 93 |
|  | JACCIA010000003.1 | 19,964,337 | 19,963,951-19,964,337 | TTATTGGG | 387 |
|  | JACCIA010000005.1 | 14,251,151 | 1-236 | TTATTGGG | 236 |
|  | JACCIA010000017.1 | 2,569,513 | 1-171 | TTATTGGG | 171 |
|  | JACCIA010000104.1 | 32,060 | 1-74 | TTATTGGG | 74 |
| *Sycobia sp.* | JACCIC010000018.1 | 5,281,343 | 5,281,140-5,281,242 | TTATTGGG | 103 |
|  | JACCIC010000033.1 | 3,993,151 | 3,991,918-3,992,892 | TTATTGGG | 975 |
|  | JACCIC010000229.1 | 817,812 | 815,027-817,212 | TTATTGGG | 2,186 |
