# Supplementary material for: Profiles of telomeric repeats in Insecta reveal diverse forms of telomeric motifs in Hymenopterans
Source: Life Sci Alliance. 2022 Apr 1;5(7):e202101163. doi: 10.26508/lsa.202101163 (PMC8977481; doi:10.26508/lsa.202101163)
Supplement: Supplementary file 9 [file LSA-2021-01163_TableS9.docx]

**Table S9. TRM calls for four parasitoid wasps species from chromosome level genome assembly.**

| **Species** | **Chromosome** | **Chromosome size (bp)** | **TRM region** | **TRM unit** | **TRM total length (bp)** |
| --- | --- | --- | --- | --- | --- |
| *Nasonia giraulti* | chr1_end | 53,945,465 | 53,943,093-53,945,465 | TTATTGGG | 2,373 |
|  | chr2_start | 45,600,855 | 1-2,695 | TTATTGGG | 2,695 |
|  | chr2_end | 45,600,855 | 45,599,229-45,600,855 | TTATTGGG | 1,627 |
|  | chr3_start | 48,325,230 | 1-2,060 | TTATTGGG | 2,060 |
|  | chr3_end | 48,325,230 | 42,323,388-48,325,230 | TTATTGGG | 1,843 |
|  | chr4_start | 56,085,809 | 1-1,822 | TTATTGGG | 1,822 |
|  | chr4_end | 56,085,809 | 56,084,164-56,085,809 | TTATTGGG | 1,646 |
|  | chr5_end | 44,664,390 | 44,662,418-44,664,390 | TTATTGGG | 1,973 |
| *Muscidifurax raptorellus* | chr1_start | 47,660,052 | 1-42 | TTATTGGG | 42 |
|  | chr1_end | 47,660,052 | 47,654,289-47,660,052 | TTATTGGG | 5,764 |
|  | chr2_start | 43,226,643 | 1-644 | TTATTGGG | 644 |
|  | chr2_end | 43,226,643 | 43,221,755-43,226,643 | TTATTGGG | 4,889 |
|  | chr3_start | 34,630,039 | 1-6,462 | TTATTGGG | 6,462 |
|  | chr3_end | 34,630,039 | 34,624,854-34,630,039 | TTATTGGG | 5,186 |
|  | chr4_start | 42,769,124 | 1-5,993 | TTATTGGG | 5,993 |
|  | chr4_end | 42,769,124 | 42,769,050-42,769,124 | TTATTGGG | 75 |
|  | chr5_start | 35,143,711 | 1-4,526 | TTATTGGG | 4,526 |
|  | chr5_end | 35,143,711 | 35,143,428-35,143,711 | TTATTGGG | 284 |
| *Muscidifurax zaraptor* | chr1_start | 48,879,205 | 1-5,799 | TTATTGGG | 5,799 |
|  | chr1_end | 48,879,205 | 48,872,552-48,879,205 | TTATTGGG | 6,654 |
|  | chr2_start | 41,893,578 | 1-3,046 | TTATTGGG | 3,046 |
|  | chr2_end | 41,893,578 | 41,887,658-41,893,578 | TTATTGGG | 5,921 |
|  | chr3_start | 37,302,792 | 1-4,011 | TTATTGGG | 4,011 |
|  | chr3_end | 37,302,792 | 37,300,262-37,302,792 | TTATTGGG | 2,531 |
|  | chr4_start | 46,372,107 | 1-5,782 | TTATTGGG | 5,782 |
|  | chr4_end | 46,372,107 | 46,365,403-46,372,107 | TTATTGGG | 6,705 |
|  | chr5_start | 32,616,567 | 1-5,144 | TTATTGGG | 5,144 |
|  | chr5_end | 32,616,567 | 32,610,525-32,616,567 | TTATTGGG | 6,043 |
| *Muscidifurax uniraptor* | chr1_start | 46,591,528 | 1-2,827 | TTATTGGG | 2,827 |
|  | chr1_end | 46,591,528 | 46,587,272-46,591,528 | TTATTGGG | 4,257 |
|  | chr2_start | 41,196,392 | 1-3,470 | TTATTGGG | 3,470 |
|  | chr2_end | 41,196,392 | 41,194,043-41,196,392 | TTATTGGG | 2,350 |
|  | chr3_start | 38,079,147 | 1-4,328 | TTATTGGG | 4,328 |
|  | chr3_end | 38,079,147 | 38,076,341-38,079,147 | TTATTGGG | 2,807 |
|  | chr4_start | 56,503,284 | 1-2,798 | TTATTGGG | 2,798 |
|  | chr4_end | 56,503,284 | 56,500,191-56,503,284 | TTATTGGG | 3,094 |
|  | chr5_start | 32,401,764 | 1-3,406 | TTATTGGG | 3,406 |
|  | chr5_end | 32,401,764 | 32,399,347-32,401,764 | TTATTGGG | 2,418 |
