# Supplementary material for: Profiles of telomeric repeats in Insecta reveal diverse forms of telomeric motifs in Hymenopterans
Source: Life Sci Alliance. 2022 Apr 1;5(7):e202101163. doi: 10.26508/lsa.202101163 (PMC8977481; doi:10.26508/lsa.202101163)
Supplement: Supplementary file 10 [file LSA-2021-01163_TableS10.docx]

**Table S10. Tandem repeat statistics computed in TRIP.**

| **Parameters** | **Definition** | **Formula** |
| --- | --- | --- |
| rpt_reads_num | the number of repeat-containing reads | return by RepeatMaster |
| total_reads_num | the number of total sequenced reads | return by RepeatMaster |
| repeats_num | the number of repeats | return by RepeatMaster |
| total_bases_num | the number of total processed bases | return by RepeatMaster |
| unit_len | the length of a repeat unit (bp) |  |
| eff_read_len | the effective length of reads (bp) | $\frac{total\_bases\_num}{total\_reads\_num}$ |
| genome_size | genome assembly length (bp) | public assembly data |
| avg_genome_cov | the average coverage of a genome | $\frac{total\_bases\_num}{genome\_size}$ |
| repeats_len | the length of repeats (bp) | $repeats\_num\cdot unit\_len$ |
| repeats_per_read | the average number of repeats per read in repeats containing reads | $\frac{repeats\_num}{rpt\_reads\_num}$ |
| reads_per_genome | the number of repeats containing reads per genome | $\frac{rpt\_reads\_num}{avg\_genome\_cov}$ |
| repeats_per_genome | number of repeats per haploid genome | $\frac{repeats\_num}{avg\_genome\_cov}$ |
| repeats_per_million_reads | number of repeats per 1 million repeats containing reads | $\frac{repeats\_num\cdot1,000,000}{rpt\_reads\_num}$ |
| repeats_len_per_genome | total repeat length (Kb) in a haploid genome | $\frac{repeats\_len}{avg\_genome\_cov\cdot1000}$ |
| repeats_len_per_million_reads | the average repeat length (Kb) per million reads | $\frac{repeats\_len\cdot1,000,000}{rpt\_reads\_num\cdot1000}$ |
| percent_repeats_len_per_read | the average percentage of repeat length in repeat-containing reads | $\frac{repeats\_len}{rpt\_reads\_num\cdot eff\_read\_len}$ |
| percent_repeats_len_per_genome | the percentage of total repeats length in a haploid genome | $\frac{repeats\_len\_per\_genome\cdot1000}{genome\_size}$ |
| percent_repeat_unit_in_seqs | the average percentage of repeat units length in all sequencing reads | $\frac{repeats\_len}{total\_bases\_num}$ |
| best_candidate_enrichment | the length ratio of the most abundant repeat over the next abundant repeat | $\frac{repeats\_len_{1st TR motif candidate}}{repeats\_len_{2nd TR motif candidate}}$ |
